# Tetrandrine Prevents Neomycin-Induced Ototoxicity by Promoting Steroid Biosynthesis

**DOI:** 10.3389/fbioe.2022.876237

**Published:** 2022-04-20

**Authors:** Qilei Zhang, Yunhao Wu, Yan Yu, Yuguang Niu, Qiaojun Fang, Xin Chen, Jieyu Qi, Chen Zhang, Geping Wu, Kaiming Su, Renjie Chai

**Affiliations:** ^1^ The Affiliated Zhangjiagang Hospital of Soochow University, Zhangjiagang, China; ^2^ State Key Laboratory of Bioelectronics, Department of Otolaryngology Head and Neck Surgery, Zhongda Hospital, School of Life Sciences and Technology, Advanced Institute for Life and Health, Jiangsu Province High-Tech Key Laboratory for Bio-Medical Research, Southeast University, Nanjing, China; ^3^ Department of Ambulatory Medicine, the First Medical Center of PLA General Hospital, Beijing, China; ^4^ Department of Neurobiology, School of Basic Medical Sciences, Beijing Key Laboratory of Neural Regeneration and Repair, Advanced Innovation Center for Human Brain Protection, Capital Medical University, Beijing, China; ^5^ Department of Otolaryngology-Head and Neck Surgery, Shanghai Jiao Tong University Affiliated Sixth People’s Hospital, Shanghai, China; ^6^ Department of Otolaryngology Head and Neck Surgery, Sichuan Provincial People’s Hospital, University of Electronic Science and Technology of China, Chengdu, China; ^7^ Co-Innovation Center of Neuroregeneration, Nantong University, Nantong, China; ^8^ Institute for Stem Cell and Regeneration, Chinese Academy of Science, Beijing, China; ^9^ Beijing Key Laboratory of Neural Regeneration and Repair, Capital Medical University, Beijing, China

**Keywords:** tetrandrine, hair cell, apoptosis, oxidative stress, steroid biosynthesis

## Abstract

Aminoglycoside antibiotics are widely used for the treatment of serious acute infections, life-threatening sepsis, and *tuberculosis*, but all aminoglycosides cause side effects, especially irreversible ototoxicity. The mechanisms underlying the ototoxicity of aminoglycosides need further investigation, and there are no effective drugs in the clinic. Here we showed that tetrandrine (TET), a bioactive bisbenzylisoquinoline alkaloid derived from *Stephania tetrandra*, ameliorated neomycin-induced cochlear hair cell injury. In both *in vitro* and *in vivo* experiments we found that TET administration significantly improved auditory function and reduced hair cell damage after neomycin exposure. In addition, we observed that TET could significantly decrease oxidative stress and apoptosis in hair cells after neomycin exposure. Finally, RNA-seq analysis suggested that TET protected against neomycin-induced ototoxicity mainly by promoting steroid biosynthesis. Collectively, our results provide pharmacological evidence showing that TET may be a promising agent in preventing aminoglycosides-induced ototoxicity.

## Introduction

Sensorineural hearing loss (SHL), results from degeneration of the sensory hair cells in the organ of Corti, and the loss of the ability to communicate with others severely impacts people’s quality of life. Aminoglycoside antibiotics, the most commonly used ototoxic drugs, are one of the leading contributors to SHL (O'Sullivan et al., 2017). Despite this, aminoglycosides are still widely used in the treatment of many diseases due to their special antibacterial activities and physicochemical properties. It is therefore of great clinical significance to explore the molecular mechanisms through which aminoglycosides damage cochlear hair cells and to develop therapeutic drugs for preventing SHL.

**FIGURE 1:**
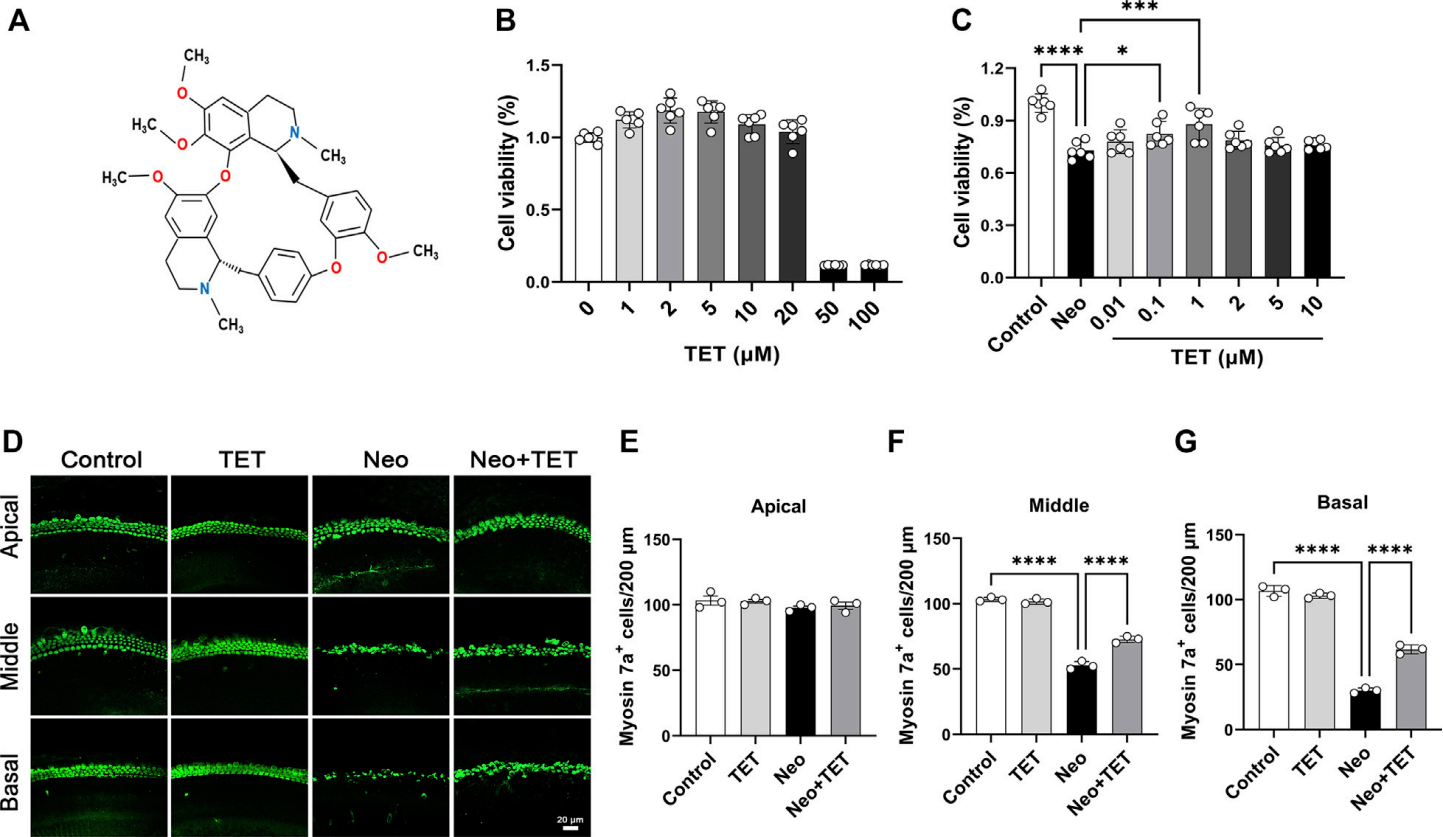
TET increases HEI-OC1 cell viability and promotes cochlear hair cell survival after neomycin exposure **(A)** Structure of TET **(B)** The toxicity of TET at different doses (1, 2, 5, 10, 20, 50, 100 μM) in HEI-OC1 cells was measured by CCK-8 (n = 6) **(C)** CCK-8 results showing the protective effects of TET on HEI-OC1 cells after neomycin treatment (n = 6) **(D)** Immunostaining of hair cells with anti-Myosin 7a antibody in the apical, middle and basal turns of the cochlea. Scale bars: 20 μm **(E-G)** Quantification of the number of cochlear hair cells per 200 μm in the apical, middle and basal turns (n = 3). *p < 0.05, ***p < 0.001, ****p < 0.0001.

Recently, several active ingredients derived from traditional Chinese medicine have shown promising otoprotective effects against SHL and tinnitus (Zhai et al., 2013; Fetoni et al., 2015; Li et al., 2019; Di et al., 2020). Tetrandrine (TET) (Figure 1A) is a natural bisbenzylisoquinoline alkaloid isolated from the roots of *Stephania tetrandra* S. Moore of the Menispermaceae family (Bhagya and Chandrashekar, 2016; Luan et al., 2020). Numerous studies have verified that TET possesses a broad-spectrum of distinct pharmacological activities such as anti-inflammatory (Ren et al., 2021), neuroprotective (Chen et al., 2016), calcium antagonistic (Ding et al., 2021), anti-hypertensive (Huang et al., 2016), and antineoplastic (Zhang et al., 2020) activities. Furthermore, it has been reported that TET has an otoprotective effect against noise-induced hearing loss (Yu et al., 2018). We thus speculated that TET may have great potential in the prevention of aminoglycoside-induced SHL.

In the present study, we aimed to investigate the protective effect of TET on neomycin-induced SHL by establishing *in vitro*, *in vivo*, and whole-organ explant culture models and contributed to the development of preventive and therapeutic drugs that protect against aminoglycosides-induced SHL.

## Results

### TET Protects Against Neomycin-Induced HEI-OC1 Damage

To determine the appropriate dose of TET in the HEI-OC1 auditory cell line prior to neomycin stimulation, the cells were treated with different concentrations of TET (1, 2, 5, 10, 20, 50 and 100 μM). The CCK8 results indicated that there was no toxicity in the range of 1–20 μM (Figure 1B). We then pretreated the HEI-OC1 cells with TET (0.01, 0.1, 1, 2, 5 and 10 μM) for 24 h and then treated the cells with 2 mM neomycin together with TET for another 24 h. We observed that TET significantly enhanced cell viability after neomycin exposure at doses of 0.1 and 1 μM (Figure 1C), so we chose 1 μM TET administration for 24 h as the optimum treatment condition. We next investigated the effect of TET on cochlear hair cell loss induced by neomycin in whole-organ explant cultures *in vitro*. The cochlear explants were pre-treated with 1 μM TET for 12 h and then treated with 0.5 mM neomycin for 12 h. Immunostaining results showed that TET markedly prevented neomycin-induced hair cell loss in the middle and basal turns of the cochlear explants (Figures 1D–G). Taken together, these results suggest that TET has protective effects against neomycin-induced hair cell damage.

### TET Attenuates Neomycin-Induced SHL *in vivo*

**FIGURE 2:**
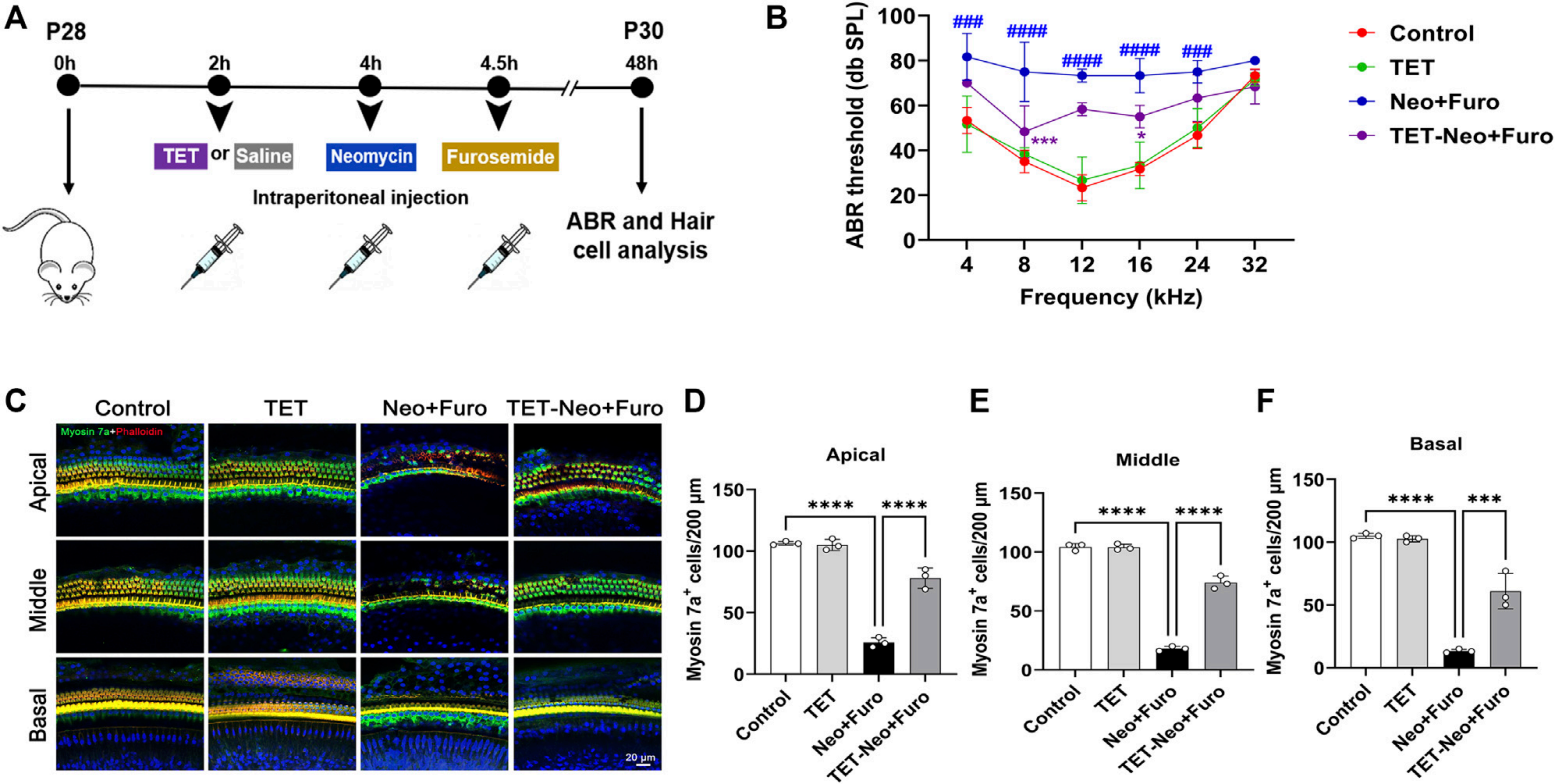
TET improves auditory function and protects against neomycin-induced cochlear hair cell loss **(A)** Schematic illustration of the acute neomycin damage model **(B)** ABR thresholds were analyzed to determine the effect of TET on neomycin-induced hearing loss in mice (n = 6) **(C)** Immunostaining of cochlear hair cells marked by myosin 7a in the apical, middle and basal turns. Scale bars: 20 μm **(D-F)** Quantification of the number of cochlear hair cells per 200 μm in apical, middle and basal turns (n = 3). *p < 0.05, ***p < 0.001, ****p < 0.0001.

To investigate the protective effects of TET on neomycin-induced SHL *in vivo*, we established an acute neomycin damage model as previously reported (He et al., 2020). Postnatal day (P)28 wild type C57 mice were injected with TET (150mg/kg) intraperitoneally, and 2 h later the mice were given neomycin (100 mg/kg) in conjunction with intraperitoneal injection of furosemide (200 mg/kg) (Figure 2A). Mice treated with TET alone and saline alone were used as controls. Auditory brainstem response (ABR) analysis showed that TET had a protective effect against neomycin-induced hearing loss and showed significant reduced threshold elevation at 8 and 16 kHz after neomycin treatment (Figure 2B). The hair cells in the cochleae were stained with myosin 7a and phalloidin. We observed that neomycin with furosemide led to massive hair cell loss in the apical, middle, and basal turns, while co-administration of TET clearly promoted hair cell survival (Figure 2C–F). Together, these results indicate that TET may attenuate neomycin-induced SHL *in vivo*.

### TET Alleviates Neomycin-Induced HEI-OC1 Cell Apoptosis

**FIGURE 3:**
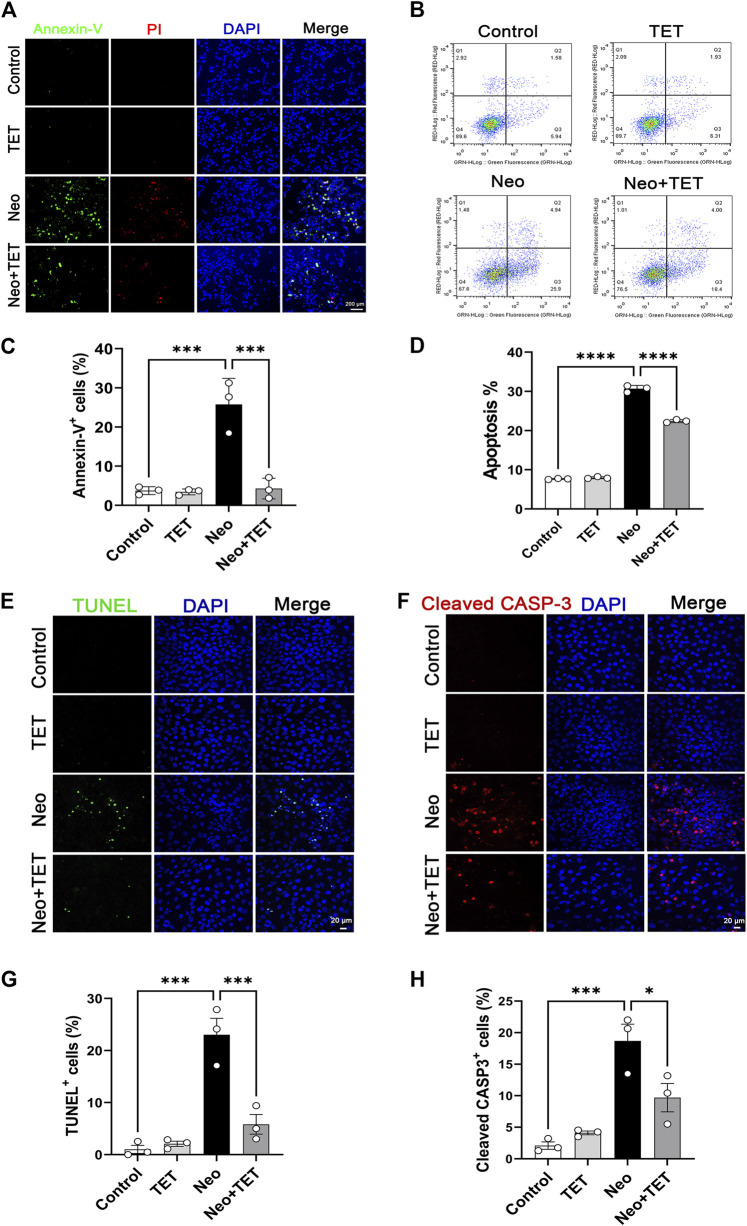
Effects of TET on neomycin-stimulated apoptosis in HEI-OC1 cells **(A)** Annexin-V/PI staining in HEI-OC1 cells after TET treatment followed by neomycin stimulation. Scale bars: 200 μm **(B)** Apoptosis analysis of HEI-OC1 cells by flow cytometry **(C)** Quantification of the numbers of Annexin-V-positive HEI-OC1 cells in **(A)** (n = 3) **(D)** Quantification of the proportions of apoptotic cells (n = 3) **(E,F)** TUNEL and cleaved caspase-3 staining of HEI-OC1 cells after TET administration followed by neomycin exposure. Scale bars: 20 μm **(G,H)** Quantification of the numbers of TUNEL/cleaved caspase-3-positive HEI-OC1 cells in **(E,F)** (n = 3). *p < 0.05, ***p < 0.001, ****p < 0.0001.

Annexin V-FITC/PI staining was used to study the protective effect of TET on neomycin-induced cell death and apoptosis in HEI-OC1 cells. Cells were pre-treated with 1 μM TET for 24 h followed by neomycin stimulation together with 1 μM TET for another 24 h. The immunofluorescence and flow cytometry analysis showed that neomycin exposure led to significant cell death and apoptosis, while TET treatment showed a remarkable improvement in cell death and apoptosis in HEI-OC1 cells (Figure 3A–D). TUNEL and cleaved caspase-3 staining were also used for the detection of apoptosis in HEI-OC1 cells. And we found that there were more TUNEL-positive and cleaved caspase-3-positive cells in the neomycin treatment group compared with the control group and that TET administration significantly reduced the proportions of TUNEL-positive and cleaved caspase-3-positive cells after neomycin treatment (Figure 3E–H). Collectively, these results disclosed that TET might prevent HEI-OC1 apoptosis and cell death when challenged by neomycin.

### TET Decreases Cochlear Hair Cell Apoptosis Induced by Neomycin

**FIGURE 4:**
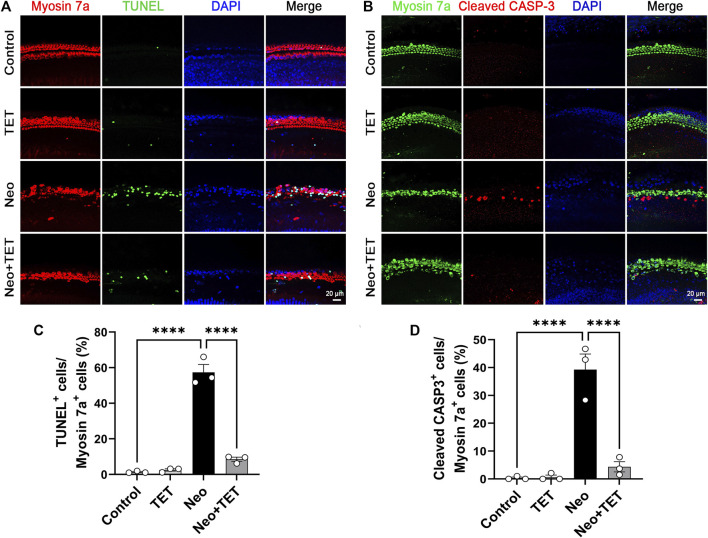
Effects of TET on hair cell apoptosis in the mouse cochlea after neomycin exposure **(A)** Immunofluorescence staining with TUNEL and myosin 7a in cochlear hair cells in the middle turn after TET treatment followed by neomycin exposure. Scale bars: 20 μm **(B)** Immunofluorescence staining with cleaved caspase-3 and myosin 7a in cochlear hair cells in the middle turn after TET treatment followed by neomycin induction. Scale bars: 20 μm **(C)** Quantification of the numbers of TUNEL/myosin 7a double-positive cells in **(A)** (n = 3) **(D)** Quantification of the numbers of cleaved caspase-3/myosin 7a double-positive cells in **(B)** (n = 3). ****p < 0.0001.

We also investigate the protective effects of TET on neomycin-induced hair cell apoptosis in whole-organ explant cultures *in vitro*. Consistent with the results discussed above, immunofluorescence staining of TUNEL/myosin 7a and cleaved caspase-3/myosin 7a showed that the numbers and proportions of TUNEL/myosin 7a double-positive and cleaved caspase-3/myosin 7a double-positive hair cells per 200 μm cochlear length of the middle turn in the neomycin treatment group were notably greater than the normal control group, while TET pre-treatment dramatically reduced the numbers and proportions of TUNEL/myosin 7a double-positive and cleaved caspase-3/myosin 7a double-positive hair cells compared with the neomycin only group (Figure 4A–D). These results indicated that pre-treatment with TET can suppress the apoptotic cascade induced by aminoglycosides.

### TET Decreases Reactive Oxygen Species (ROS) Production After Neomycin Treatment in HEI-OC1 Cells

**FIGURE 5:**
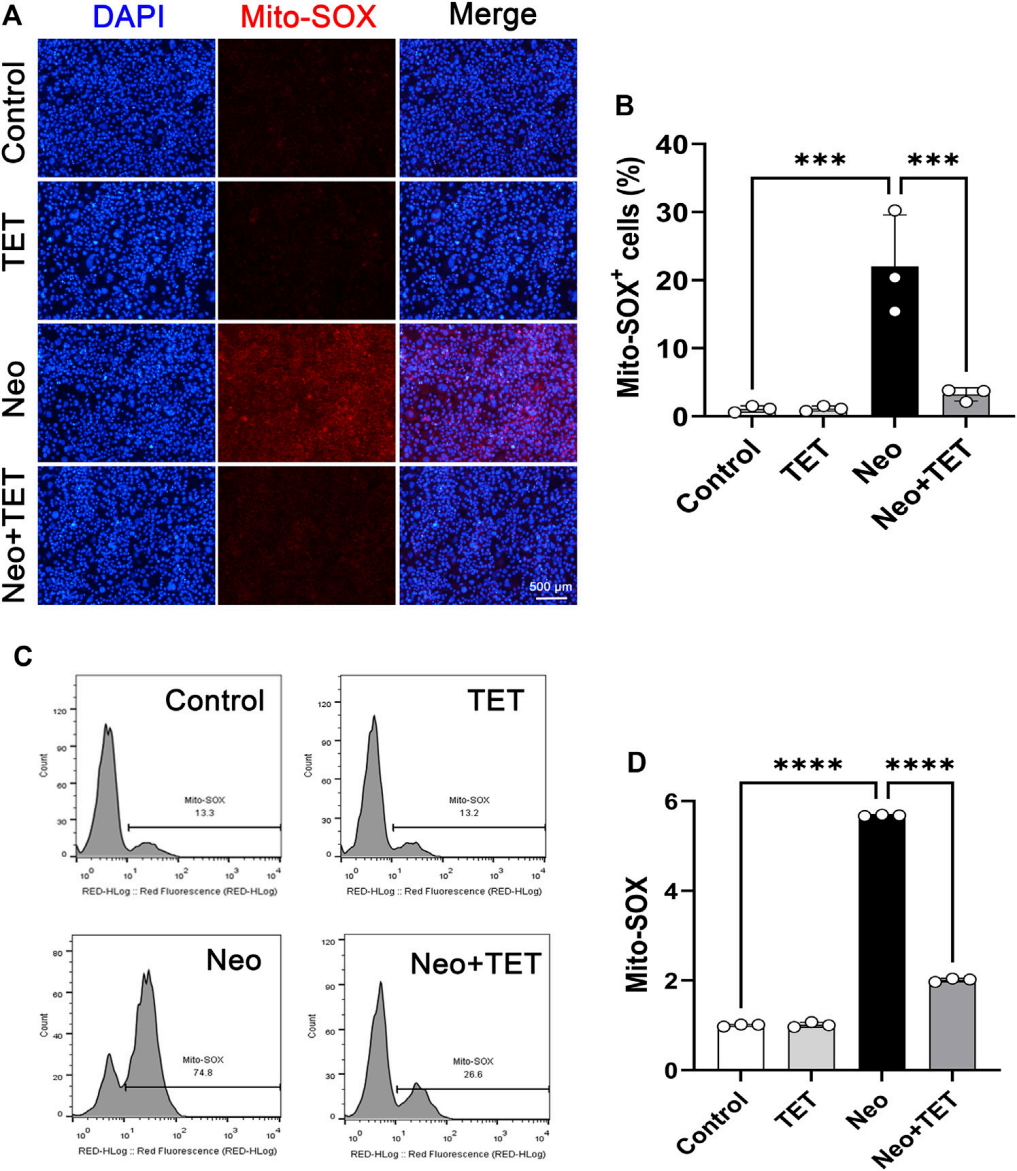
Effects of TET on neomycin-induced oxidative stress in HEI-OC1 cells **(A)** HEI-OC1 cells were labeled with a Mito-SOX staining kit (n = 3). Scale bars: 500 μm **(B)** Quantification of the numbers of Mito-SOX-positive HEI-OC1 cells in **(A)** (n = 3) **(C)** ROS accumulation was further confirmed by flow cytometry (n = 3) **(D)** Quantification of the results of flow cytometry in **(C)**. ***p < 0.001, ****p < 0.0001.

It has been reported that the accumulation of ROS in mitochondria is the initiating contributor to drugs-induced ototoxicity (Choung et al., 2009; Schacht et al., 2012). We used a Mito-SOX™ Red kit to determine the levels of mitochondrial superoxide in HEI-OC1 cells. Immunostaining analysis showed that there were almost no Mito-SOX Red/myosin 7a double-positive cells in the normal control group and TET only group, but ROS accumulation was prominently increased after neomycin treatment for 24 h compared to the control group, and this increase was strongly inhibited by TET administration (Figure 5A,B). In accordance with the immunostaining data, flow cytometry results showed that TET could attenuate neomycin-induced oxidative stress in HEI-OC1 cells (Figure 5C,D). Together, these results suggest that TET pre-treatment might alleviate neomycin-induced oxidative stress.

### TET Prevents Neomycin-Induced Ototoxicity by Promoting Steroid Biosynthesis

**FIGURE 6:**
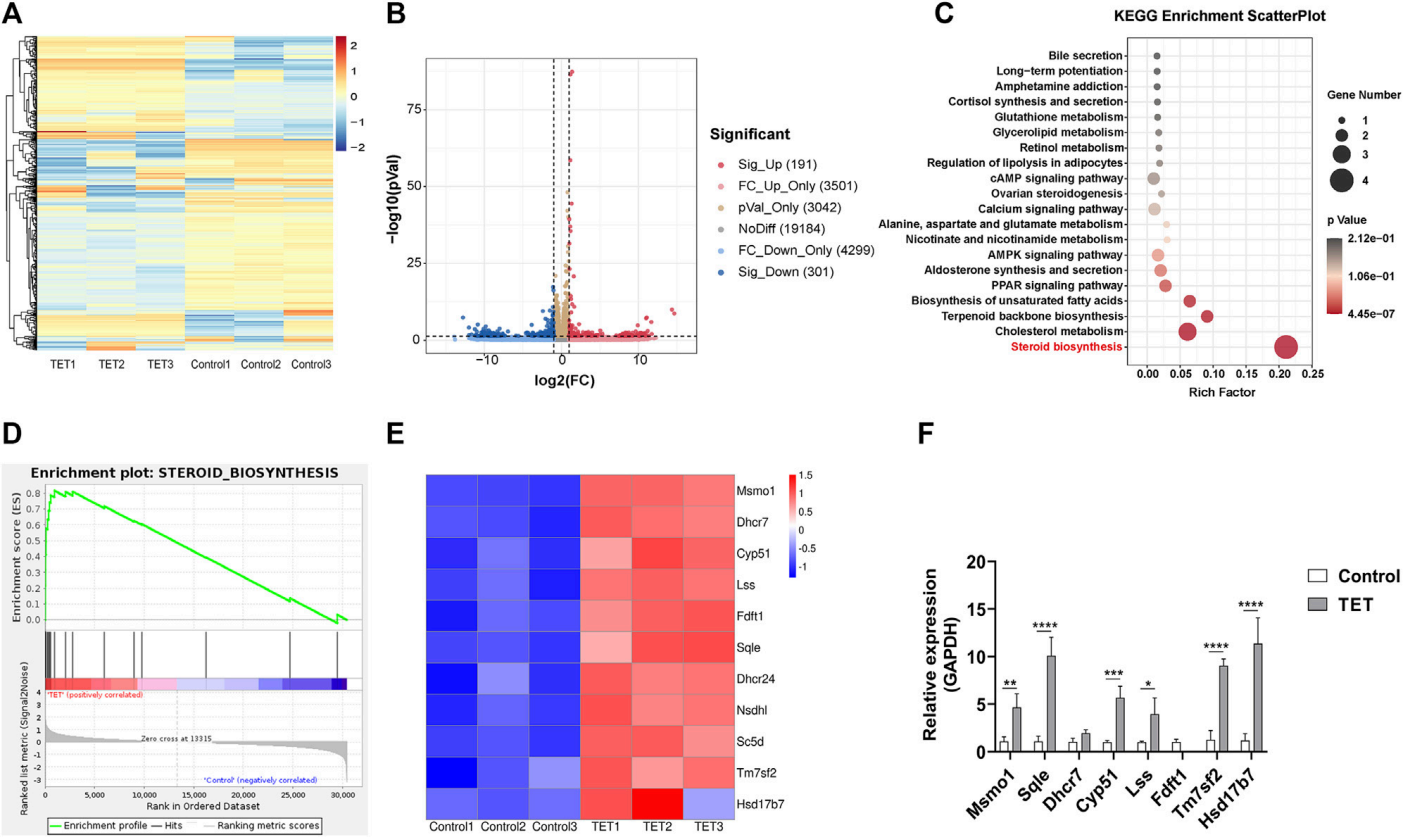
TET promotes steroid biosynthesis in the cochlea **(A)** Heat map representation of significantly differentially expressed genes after TET treatment **(B)** Volcano plot representation of significantly differentially expressed genes after TET treatment **(C)** KEGG pathway enrichment analysis of the most significantly changed pathways after TET treatment **(D)** GSEA of genes related to steroid biosynthesis in the control and TET treatment groups **(E)** Heat map of differentially expressed genes in the steroid biosynthesis pathway **(F)** Real-time PCR analysis of differentially expressed genes related to the steroid biosynthesis pathway (n = 3). *p < 0.05, **p < 0.01, ***p < 0.001, ****p < 0.0001.

To further explore the possible underlying mechanism of TET on neomycin-induced ototoxicity, the extracted RNA of cochlear explants was subjected to RNA sequencing. After TET treatment, there were 492 significantly differentially expressed genes, including 191 up-regulated genes and 301 down-regulated genes (Figure 6A,B). To identify the pathways involved in TET regulation, we performed pathway enrichment analysis based on the Kyoto Encyclopedia of Genes and Genomes (KEGG) database. Among the top significantly enriched signaling pathways, steroid biosynthesis was the most significantly enriched functional pathway in the TET treatment group (Figure 6C). To further validate our data, gene set enrichment analysis (GSEA) was applied to the differentially expressed genes between the control and TET treatment group. This analysis confirmed that steroid biosynthesis was significantly enriched in the TET treatment group (Figure 6D). The significantly changed genes that were enriched in steroid biosynthesis were mainly involved in cholesterol biosynthesis (*Msmo1*, *Lss*, *Fdft1*, *Dhcr24*, *Sc5d*, *Tm7sf2*, and *Hsd17b7*), cholesterol metabolism (*Fdft1*, *Nsdhl*, and *Sqle*), isoprenoid biosynthesis (*Fdft1*) and sterol biosynthesis (*Dhcr7* and *Fdft1*) (Figure 6E). By using quantitative real-time PCR, we confirmed that the steroid biosynthesis-related mRNA expression was significantly increased in the TET treatment group compared to the control group (Figure 6F). Collectively, these results suggest that TET can prevent neomycin-induced ototoxicity mainly by promoting steroid biosynthesis.

## Materials and Methods

### Animal Experiments

C57BL/6 mice (4 weeks-old) were obtained from Vital River Laboratory Animal Technology (Beijing, China). All animal experiments were carried out according to protocols approved by the Animal Care and Use Committee of Southeast University and were consistent with the National Institutes of Health Guide for the Care and Use of Laboratory Animals.

Mice at P28 received an intraperitoneal injection of 150 mg/kg TET (Topscience, T2996), and the mice in the control group were injected with the same volume of saline. After 2 h, mice were given 100 mg/kg neomycin (Sigma, N6386) by intraperitoneal injection followed by a single dose of 200 mg/kg furosemide (Sigma, BP547) 0.5 h later. The ABR threshold detection and hair cell counting assay were performed 2 days later.

### Immunofluorescence Analysis

The dissected mouse cochleae or cultured HEI-OC1 cells were fixed in 4% paraformaldehyde. After washsing three times with 1% Triton X-100 in PBS (PBST) for 5 min each time, the samples were blocked with 10% donkey serum for 1 h at room temperature, followed by incubation with primary antibody at 4°C overnight. After washing three times with PBST, samples were incubated with secondary antibody with or without phalloidin for 1.5 h at room temperature, then washed three times again for 5 min each time and imaged under a confocal microscope.

The primary antibodies used in the present study were: myosin 7a (*Proteus* Bioscience, 25–6790, 1:1000 dilution) and cleaved caspase-3 (Cell Signaling Technology, #9661, 1:400 dilution). The secondary antibodies were: goat anti-rabbit, Alexa Fluor Plus 488 (Thermo Fisher, A32731, 1:400 dilution); goat anti-mouse, Alexa Fluor Plus 555 (Thermo Fisher, A32727, 1:400 dilution), goat anti-rabbit, Alexa Fluor Plus 555 (Thermo Fisher, A32732, 1:400 dilution), and Alexa FluorTM 594-conjugated phalloidin (Thermo Fisher, A12381, 1:400 dilution).

### Cell Treatments

HEI-OC1 cells were seeded into 96-well plates at 6 × 10^4^ cells/well for 24 h. Then the cells were treated with different concentrations of TET (0.01, 0.1, 1, 2, 5 and 10 μM) for 24 h followed by the treatment with neomycin for 24 h. CCK8 solution (10%) was added to the plates for 1 h, the absorbance at 450 nm was detected by a microplate reader.

### Cochlear Explant Culture

The cochleae from the inner ear in neonatal (P3) mice were dissected and placed in sterile Hank’s Balanced Salt Solution under a microscope. After removing the spiral ligament, stria vascularis and spiral ganglion, the cochleae were placed in a four-well dish covered with Cell-Tak (Corning, 354,240) and cultured in DMEM-F12 medium containing B-27TM (Thermo Fisher, 17504044), N2 (Thermo Fisher, 17502001) and ampicillin (Beyotime, ST008). After incubation at 37°C in a 5% CO_2_ cell incubator for 12 h, the cochleae were pre-treated with 1 μM TET for 12 h and then given 0.5 mM neomycin and 1 μM TET for another 12 h.

### ABR Audiometry

ABR was used to measure the hearing thresholds of the mice. As has been reported previously (Liu et al., 2019), anesthetized mice were placed on a thermostatic heating pad, and the hearing threshold was evaluated at six frequencies (4, 8, 12, 16, 24, and 32 kHz) on a TDT System three apparatus (Tucker Davies Technologies, Gainesville, FL, United States).

### TUNEL Staining

The samples were balanced with 1× equilibration buffer for 20 min at temperature. Then the samples were incubated with 50 μL labeling buffer which including ddH_2_O, equilibration buffer, brightgreen labeling mix and recombinant TdT enzyme at 37°C for 60 min. After washed 3 times with PBS, myosin 7a and DAPI were used as counterstain to mark hair cells. Images were obtained using a confocal microscope.

### Flow Cytometry

An Annexin V-FITC/PI (BD, 556,419) kit was used to determine the levels of apoptosis and necrosis in HEI-OC1 cells. In brief, HEI-OC1 cells were trypsinized and centrifuged at 300 ×*g* for 5 min. After washing 3 times with cold PBS, cells were incubated with FITC Annexin V in a buffer containing propidium iodide and analyzed by flow cytometry (FACSCanto, BD, San Jose, CA, United States).

A Mito-SOX kit (Thermo Fisher, M36008) was employed to measure the oxidative stress level of HEI-OC1 cells. Cells were trypsinized and collected by centrifugation at 300 *g* for 5 min. The cells were then resuspended in Mito-SOX solution for 10 min, washed three times in PBS, and analyzed by flow cytometry.

### Quantitative Real-Time PCR (qRT-PCR)

The total cochlear RNA was extracted using a FastPure Cell/Tissue Total RNA Isolation Kit (Vazyme, RC112-01) and was reversed transcribed to cDNA using a HiScript III first Strand cDNA Synthesis Kit (Vazyme, R312-01). The qRT-PCR was performed on an Applied Biosystems CFX96 real-time PCR system (Bio-Rad, Hercules, CA, United States) using the AceQ universal SYBR qPCR Master Mix (Vazyme, Q511-02). The qRT-PCR conditions were set as follows: 5 min denaturing at 95°C followed by 40 cycles at 95°C for 10 s, 60°C for 30 s, then 95°C for 15 s, 60°C for 60 s and 95°C for 15 s according to the manufacturer’s recommendations. GAPDH was used as the control and the results were calculated using the comparative cycle threshold (∆∆Ct) method.

### Statistical Analysis

The data are shown as mean ± standard deviation (SD). The GraphPad Prism 9 software was used in the data analysis. Statistical significance was calculated with one-way analysis of variance (ANOVA) followed by the Dunnett’s test. Differences were considered statistically significant at *p* values <0.05.

## Discussion

The results of the present study showed a previously unrecognized role for TET in protecting against aminoglycoside-induced ototoxicity, and the possible mechanism was investigated. We found that TET could attenuate neomycin-induced hearing loss *in vivo* and in HEI-OC1 cells and in injured cochlear explants *in vitro*. In addition, TET treatment effectively reversed neomycin-induced ROS accumulation in hair cells and prevented apoptosis in both HEI-OC1 cells and cochlear explants. Furthermore, RNA-seq analysis showed that TET treatment could significantly promote steroid biosynthesis in cochlear hair cells, which might be the main way that TET exerts its protective effects against neomycin-induced ototoxicity.

The clinical use of aminoglycosides is hampered by their ototoxic side effects. It has been reported that aminoglycosides are permeant blockers and that they can enter cochlear hair cells by interacting with several kinds of ion channels such as mechanoelectrical transducer channels (Xie et al., 2011; Kenyon et al., 2017; Kros and Steyger, 2019). Thus aminoglycosides accumulate in hair cells where they inhibit mitochondrial protein synthesis resulting in oxidative stress, and this leads to the initiation of apoptotic signaling cascades in the inner ears (Shulman et al., 2014; Esterberg et al., 2016; Desa et al., 2018). Despite multiple agents having been reported to protect against aminoglycoside-induced SHL, it is a concerning reality that none of the described agents have yet entered clinical trials. Previous research showed that TET could prevent outer hair cell damage and synapse loss after noise exposure (Yu et al., 2018), but there is little information about the effect of TET on neomycin-induced ototoxicity.

In this study we first investigated the protective effect of TET on neomycin-induced cochlear hair cell injury, and we observed that TET significantly promoted HEI-OC1 cell survival and reversed cochlear hair cell loss after neomycin treatment *in vitro* (Figure 1). To further evaluate whether TET can protect against neomycin-induced hearing loss *in vivo*, ABR analysis and cochlear hair cell counting by immunostaining were performed. These results indicated that TET had a strong protective effect on low frequency and middle frequency thresholds (8 and 16 kHz) and that it remarkably attenuated neomycin-induced cochlear hair cell loss (Figure 2).

An event that is frequently and predominantly involved in the degeneration of cochlear mechanosensory hair cells is the accumulation of toxic levels of ROS after aminoglycosides exposure (Denamur et al., 2011; Ye et al., 2018), and interventions using diverse antioxidants both *in vitro* and *in vivo* have proven to be effective in protecting against the ototoxicity of aminoglycosides (Tokgoz et al., 2011; Polony et al., 2014; Sekulic-Jablanovic et al., 2020). Overproduction of ROS may lead to the activation of caspase 3, which initiates apoptosis in hair cells (Kim et al., 2016; Wen et al., 2018). In the current study, Mito-SOX staining and flow cytometry were used to determine the antioxidative effect of TET in HEI-OC1 cells after neomycin exposure. The results indicated that TET could significantly reduce the oxidative stress level and ameliorate hair cell apoptosis after neomycin exposure (Figures 3–5).

To further explore the possible mechanism by which TET protects against neomycin-induced hair cell injury, we performed an RNA-seq analysis of cochlear explants after TET treatment. We observed a total of 191 significantly up-regulated differentially expressed genes and 301 significantly down-regulated differentially expressed genes. KEGG enrichment analysis showed that TET mainly promoted steroid biosynthesis (Figure 6). Steroid therapy is commonly used to treat sudden SHL clinically (Skarżyńska et al., 2018; Kapoor et al., 2020; Song et al., 2021), and it has been reported that steroids including corticosteroids like dexamethasone, neuroactive steroids such as dehydroepiandrosterone, and bile acids like tauroursodeoxycholic acid exert protective effects against aminoglycoside-induced cochlear hair cell damage (Nakamagoe et al., 2011; Bas et al., 2012; Jia et al., 2018). Therefore, we propose that TET attenuates neomycin-induced cochlear hair cell injury by promoting steroid biosynthesis.

## Conclusion

In summary, our results show that TET exerts a protective effect against neomycin-induced cochlear hair cell damage by reducing ROS accumulation and attenuating cell apoptosis, possibly due to the promotion of steroid biosynthesis. These findings identified an anti-oxidative and anti-apoptotic effect of TET in aminoglycoside-induced ototoxicity, and TET may therefore have clinical potential for the management of aminoglycoside-induced SHL.

## Data Availability

The original contributions presented in the study are included in the article/Supplementary Material, further inquiries can be directed to the corresponding authors.
