# Supplementary material for: Tetrandrine Prevents Neomycin-Induced Ototoxicity by Promoting Steroid Biosynthesis
Source: Front Bioeng Biotechnol. 2022 Apr 20;10:876237. doi: 10.3389/fbioe.2022.876237 (PMC9065337; doi:10.3389/fbioe.2022.876237)
Supplement: Supplementary file 13 [file DataSheet1.docx]

Tetrandrine prevents neomycin-induced ototoxicity by promoting steroid biosynthesis

**Fig. 1A**


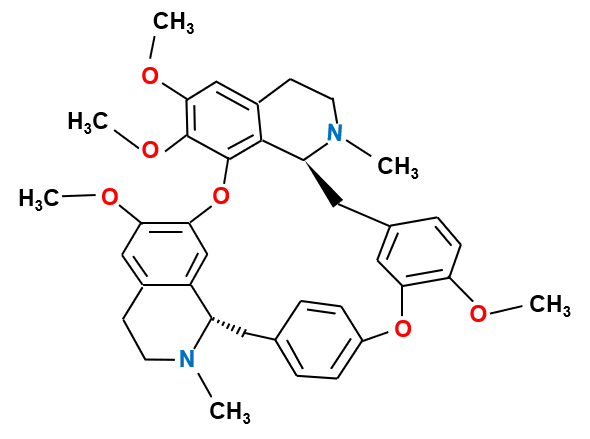


**Fig. 1D**


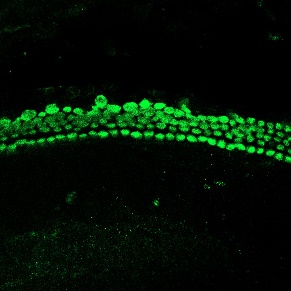

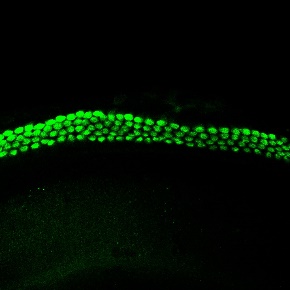

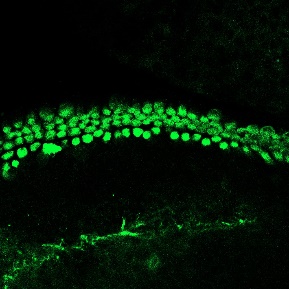

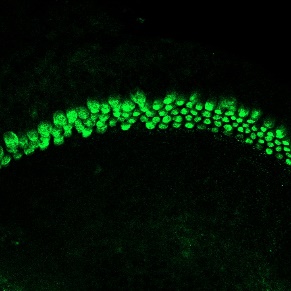

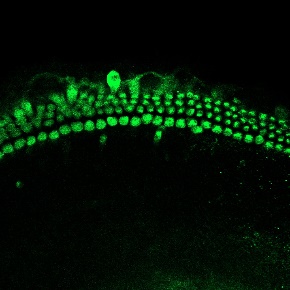

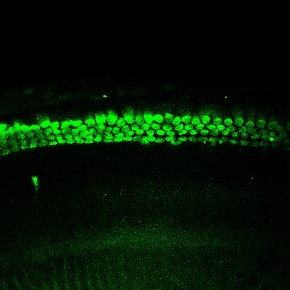

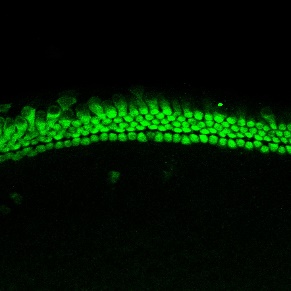

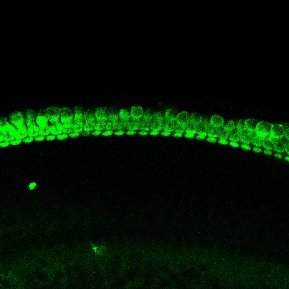

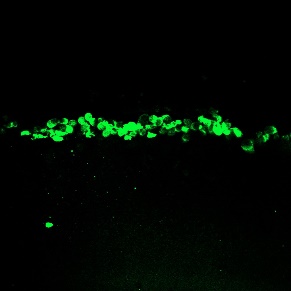

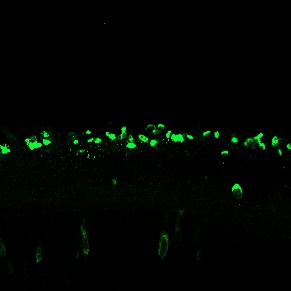

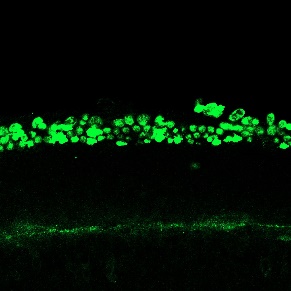

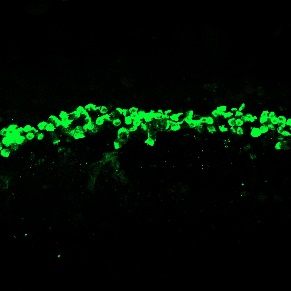


**Fig. 2A**


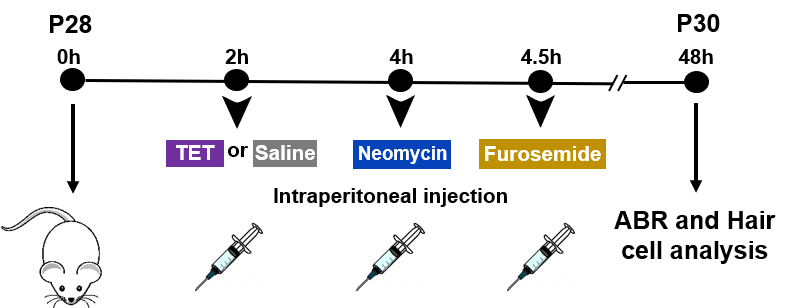


**Fig. 2C**


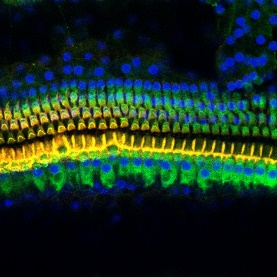

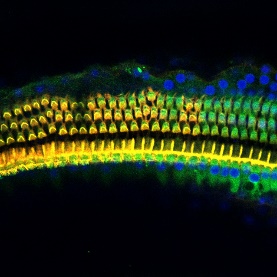

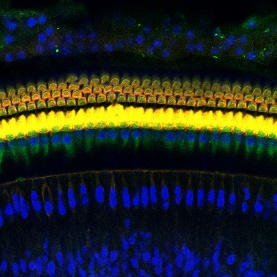

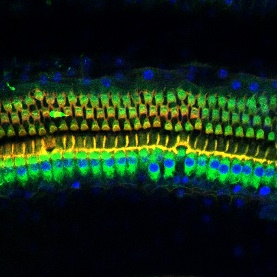

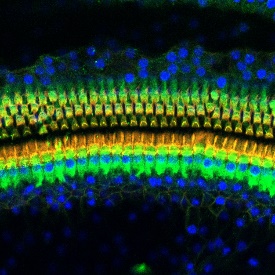

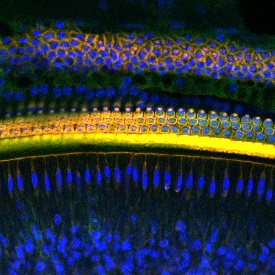

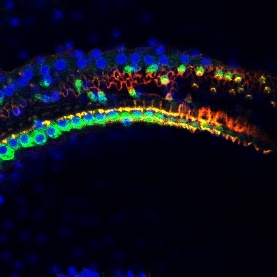

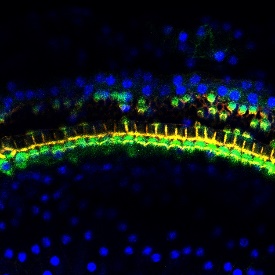

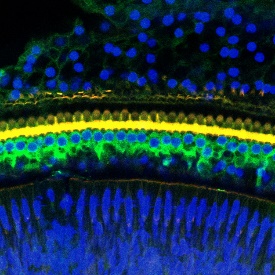

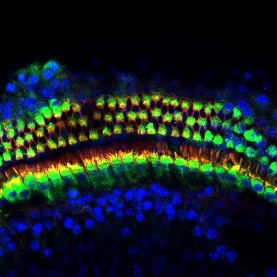

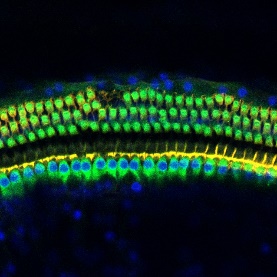

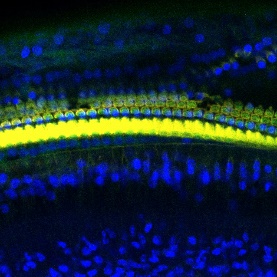


**Fig. 3A**


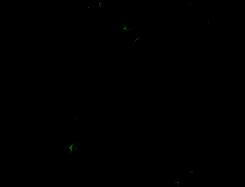

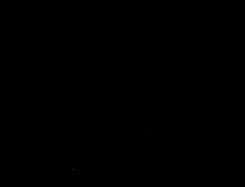

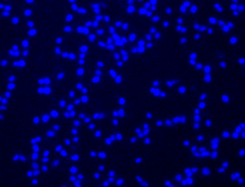

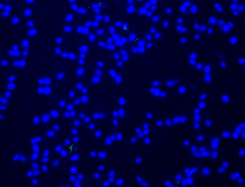

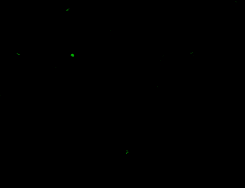

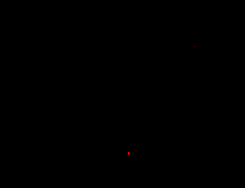

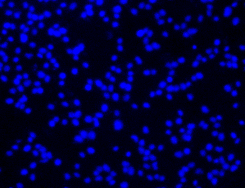

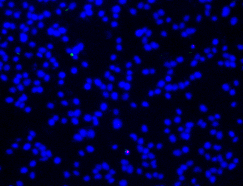

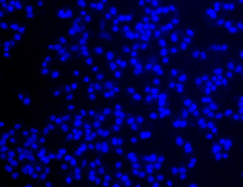

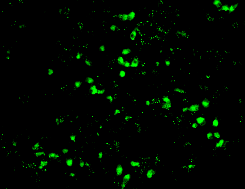

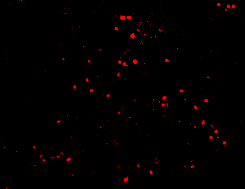

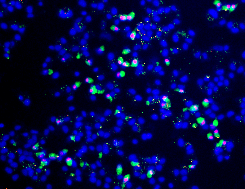

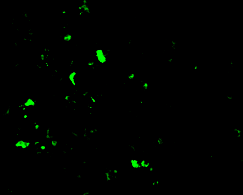

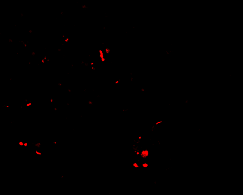

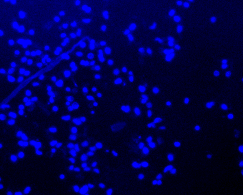

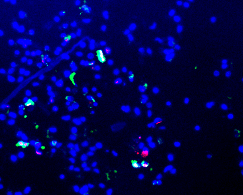


**Fig. 3B**


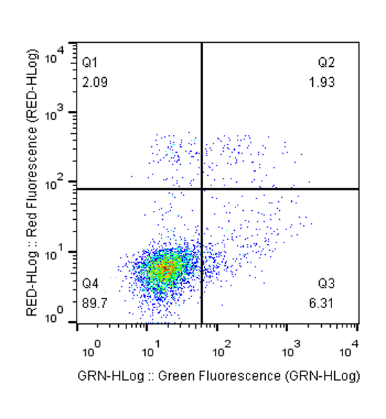

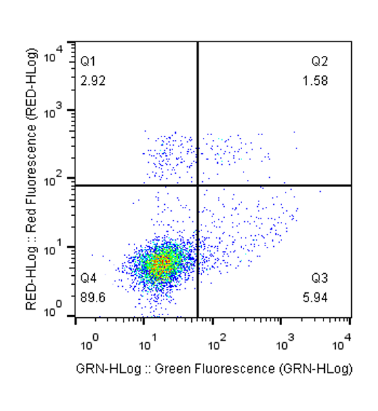


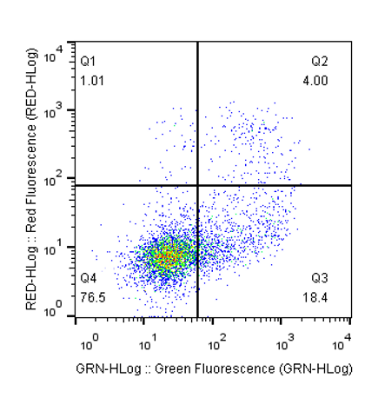

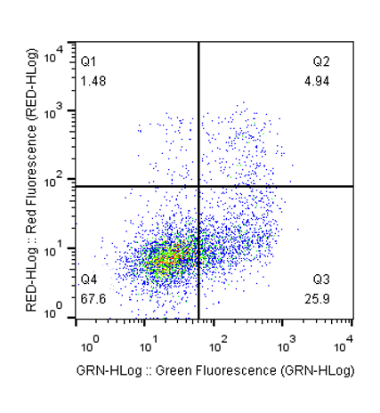


**Fig. 3D**


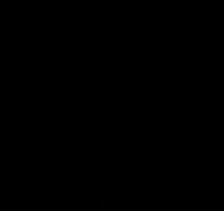

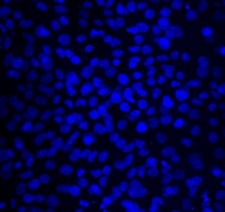

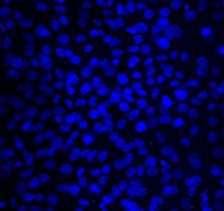

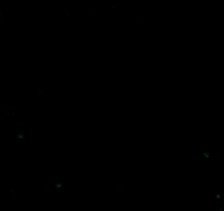

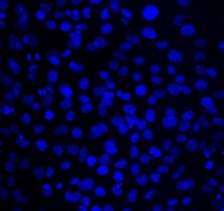

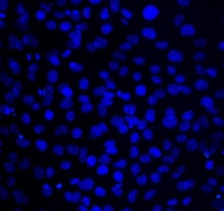

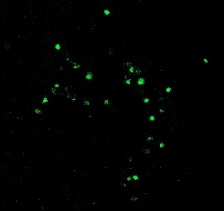

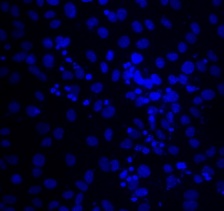

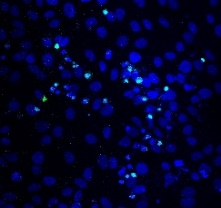

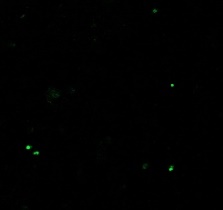

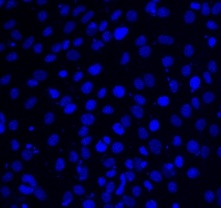

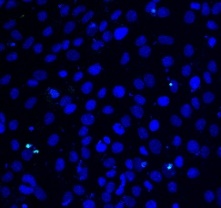


**Fig. 3F**


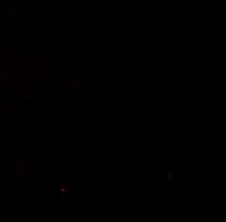

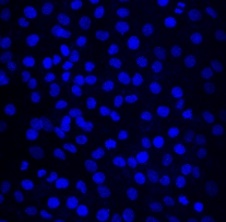

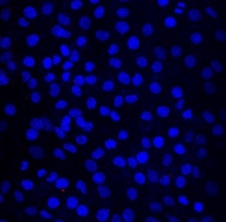

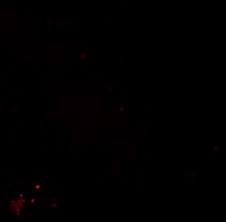

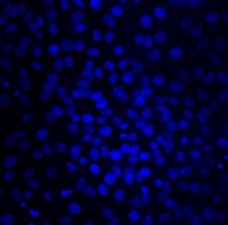

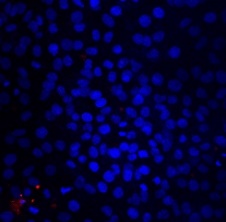

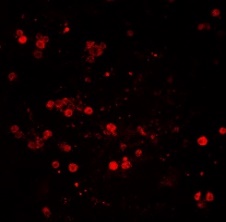

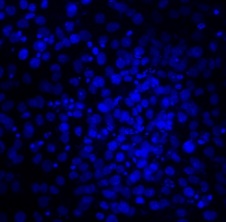

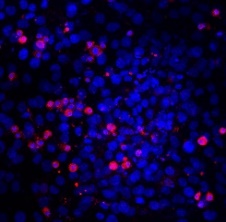

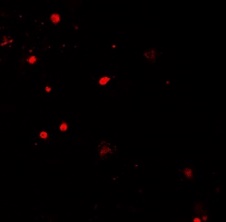

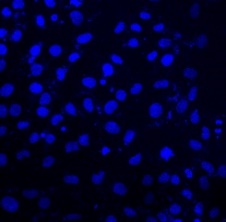

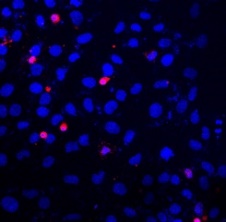


**Fig. 4A**


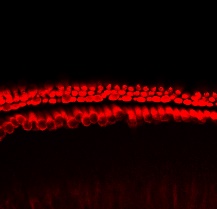

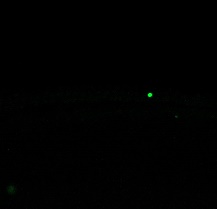

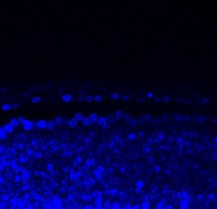

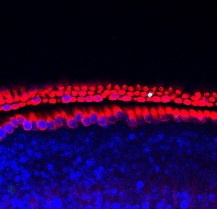

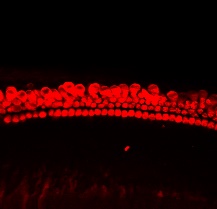

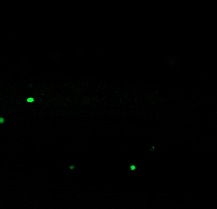

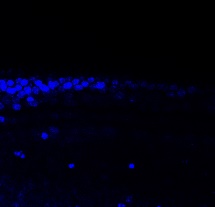

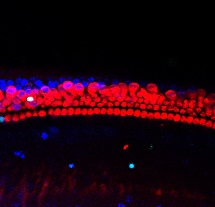

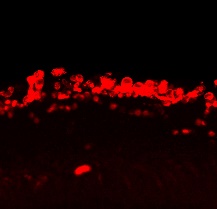

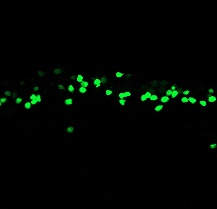

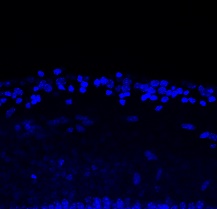

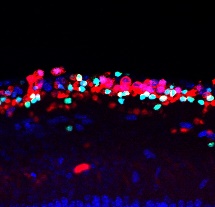

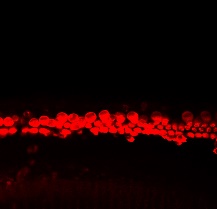

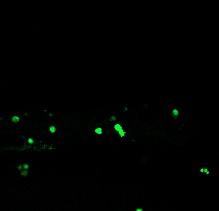

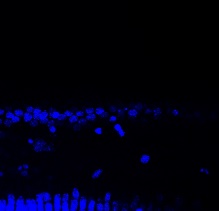

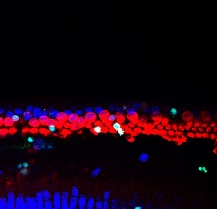


**Fig. 4B**


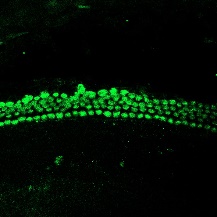

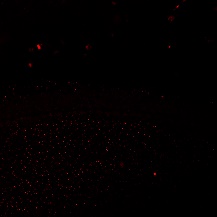

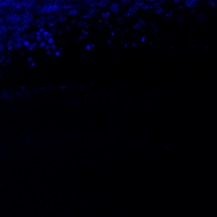

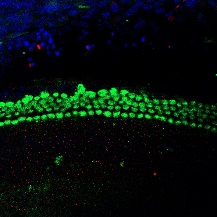

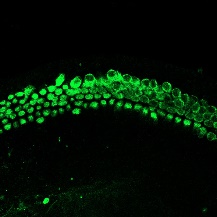

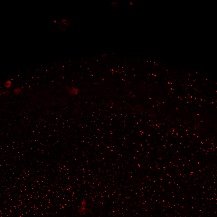

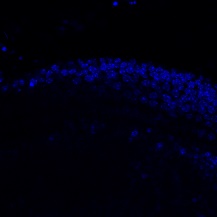

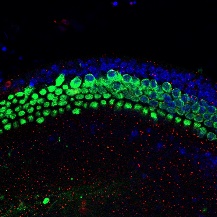

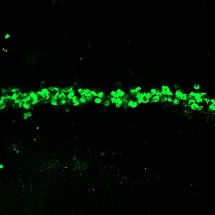

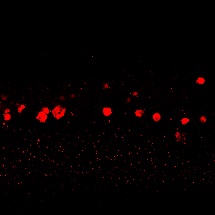

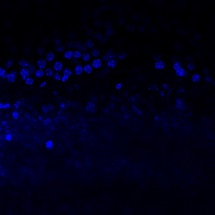

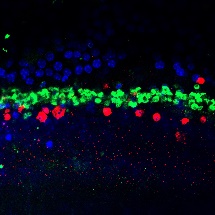

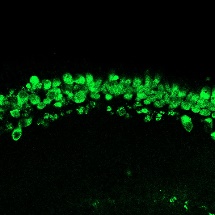

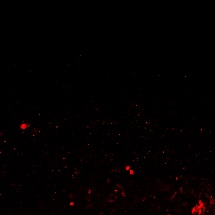


**Fig. 5A**

**Fig. 5B**

**Fig. 6A**

**Fig. 6B**

**Fig. 6C**

**Fig. 6D**

**Fig. 6E**
